# Case Report: Meningoencephalomyelitis of Unknown Etiology Manifesting as a Bilateral Cranial Polyneuropathy in 3 Dogs

**DOI:** 10.3389/fvets.2020.00326

**Published:** 2020-06-12

**Authors:** Hilary A. Levitin, Rachel Lampe, Silke Hecht

**Affiliations:** ^1^Department of Veterinary Clinical Medicine, College of Veterinary Medicine, Veterinary Urbana, University of Illinois, Urbana, IL, United States; ^2^Department of Small Animal Clinical Sciences, College of Veterinary Medicine, University of Tennessee, Knoxville, Knoxville, TN, United States

**Keywords:** MUE, MUO, cranial nerve, canine, inflammatory, brain

## Abstract

A bilateral cranial polyneuropathy was the primary magnetic resonance imaging (MRI) finding in three medium to large breed dogs diagnosed with meningoencephalomyelitis of unknown etiology. All three dogs presented with a progressive history of vestibular ataxia with either central vestibular or multifocal central nervous system (CNS) neuroanatomical localization. Brain MRI revealed variable degree of bilateral enlargement and/or increased contrast enhancement of the optic, oculomotor, trigeminal, facial, and vestibulocochlear nerves, as well as enhancement of the orbital fissure (oculomotor, trochlear, ophthalmic branch of trigeminal, and abducens nerves). There was evidence of intracranial and cranial cervical meningeal contrast enhancement in all three dogs and of cervical spinal cord lesions in 2. In all cases, more cranial nerves were affected than indicated by neurological examination. Cerebrospinal fluid (CSF) analysis was consistent with a mononuclear pleocytosis in 2 cases and a mixed cell (predominantly lymphocytic) pleocytosis in 1 case. All dogs were treated with immune suppressing medications and showed clinical improvement, although some cranial nerve deficits were persistent at follow up 2 months later. These are the first known cases of MUE diagnosed ante-mortem in a canine population documenting bilaterally symmetrical lesions affecting multiple cranial nerves. While MUE is a common cause of non-infectious inflammatory disease in dogs, it likely encompasses more classifications than have previously been reported, and should remain a differential for dogs of all ages and sizes presenting with cranial nerve deficits.

## Introduction

Meningoencephalomyelitis of unknown etiology (MUE) is a common cause of non-infectious inflammatory disease of the central nervous system in dogs (1, 2). The name MUE has been utilized as an umbrella term to describe various non-infectious inflammatory conditions, including granulomatous meningoencephalitis (GME), necrotizing meningoencephalitis (NME), necrotizing leukoencephalitis (NLE), steroid responsive meningitis arteritis, and others less common forms of the disease (2). The clinical manifestation of MUE is highly dependent upon neuroanatomic localization, most commonly including altered mentation, seizures, paresis, ataxia (including general proprioceptive, vestibular, and cerebellar), and cranial nerve deficits (1–6). Changes in vision are also possible, and may be due to a focal form of granulomatous meningoencephalitis, or a central lesion due to a multifocal MUE (7). While cranial nerve deficits have been reported in dogs with MUE, these are often due to brainstem lesions or limited to the optic nerve (which is part of the CNS). To the best of our knowledge, there are no reports of ante-mortem diagnosis of MUE manifesting as a bilateral cranial polyneuropathy. This case report describes the presentation, MRI findings, treatment, and outcome of three dogs with MUE characterized by a cranial polyneuropathy.

## Case Descriptions

### Case 1

A 10-year-old female spayed mixed breed dog (38.4 kg) was presented with a 3-day history of ataxia, weakness, facial droop, and a decreased appetite. Physical examination was unremarkable. Abnormal findings on the neurological examination included dull mentation, non-ambulatory tetraparesis with cerebellar and vestibular ataxia, an intermittent mild left head tilt, positional vertical nystagmus in both eyes (OU), right sided facial droop, in addition to absent menace response, palpebral reflex, ptosis, enophthalmos, and elevated nictitans of the right eye (OD). Postural reactions were normal in the thoracic limbs, and absent in the pelvic limbs. The withdrawal and patellar reflexes were normal. A neuroanatomical localization was assigned to the brainstem and cerebellum (central vestibular dysfunction), in addition to the right facial nerve. Differential diagnoses included auto-immune inflammatory disease (meningoencephalitis of unknown etiology), infectious disease such as Blastomycosis, Neosporosis, Toxoplasmosis, or unlikely a Rickettsial meningoencephalitis and canine distemper virus (CDV).

Clinical pathology abnormalities revealed an inflammatory leukogram (WBC: 23.57 [6–17 x 20^3^/ul]; segmented neutrophils 21.68 [3–11.5 x 10^3^]), mildly elevated liver values, and unremarkable urinalysis. An Idexx SNAP 4Dx was also performed and was negative for antibodies to Borrelia burgdorferi, Anaplasma phagocytophilum, and Ehrlichia canis, in addition to heartworm antigen. A Schirmer tear test was performed and was within the normal reference range (27 mm/min OD and 21 mm/min OS). Thoracic radiographs obtained prior to advanced imaging did not show any clinically significant findings.

Magnetic resonance imaging of the brain and the cranial cervical spine was performed using a 3T MRI system (Siemens Skyra; Siemens Medical Solutions, Malvern, PA, USA). MRI sequences included sagittal and dorsal T2-weighted (T2W), transverse T2W, T1-weighted (T1W), T2W fluid attenuation inversion recovery (T2W-FLAIR), susceptibility weighted and diffusion weighted images, and post gadolinium-based contrast T1W sequences in three planes. Contrast medium (Omniscan® GE Healthcare Inc., USA) was administered intravenously at a dose of 0.1 mmol/kg.

**Figure 1:**
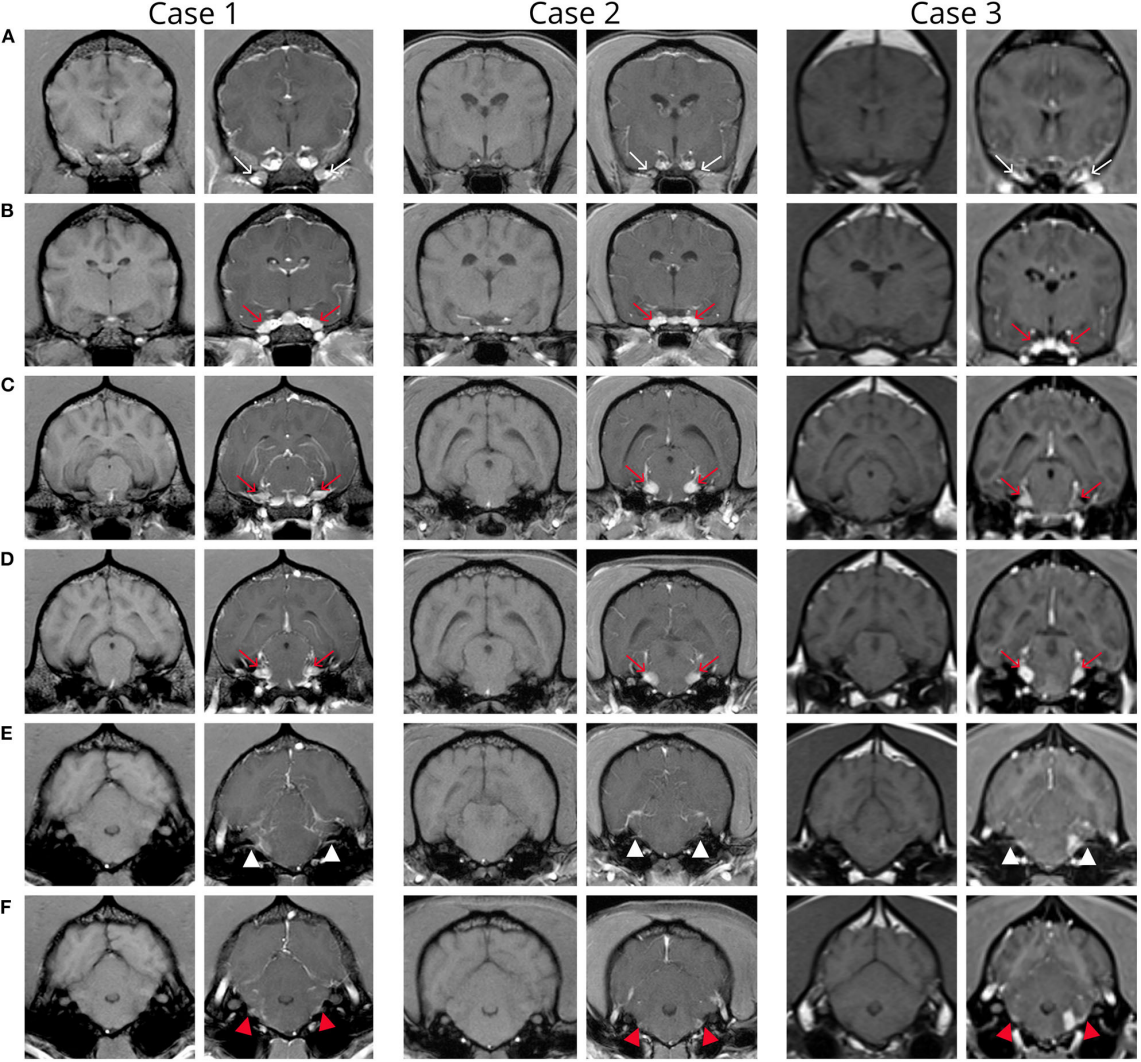
Transverse pre and post contrast T1-weighted images of the brain in patients 1-3 from rostral to caudal. Note variable severity meningeal contrast enhancement in all three patients. **(A)** Images at the level of the orbital fissure show intense contrast medium uptake within the orbital fissure (thin arrows). **(B)** Images at the level of the round foramen show increased contrast medium uptake of the maxillary branch of the trigeminal nerve bilaterally in all cases and subjective enlargement in cases 1 and 2 (red arrows). **(C,D)** Images at the level of the mesencephalon show enlargement and increased contrast enhancement of the trigeminal nerves (red arrows). **(E,F)** Images at the level of the metencephalon show increased contrast medium uptake of the facial (white arrow) and vestibulocochlear (red arrow) nerves. Note the focal left sided extraaxial lesion (corresponding to the origin of cranial nerves VII and VIII) with adjacent meningeal thickening (“dural tail sign”) in case 3 **(F)**.

**Figure 2:**
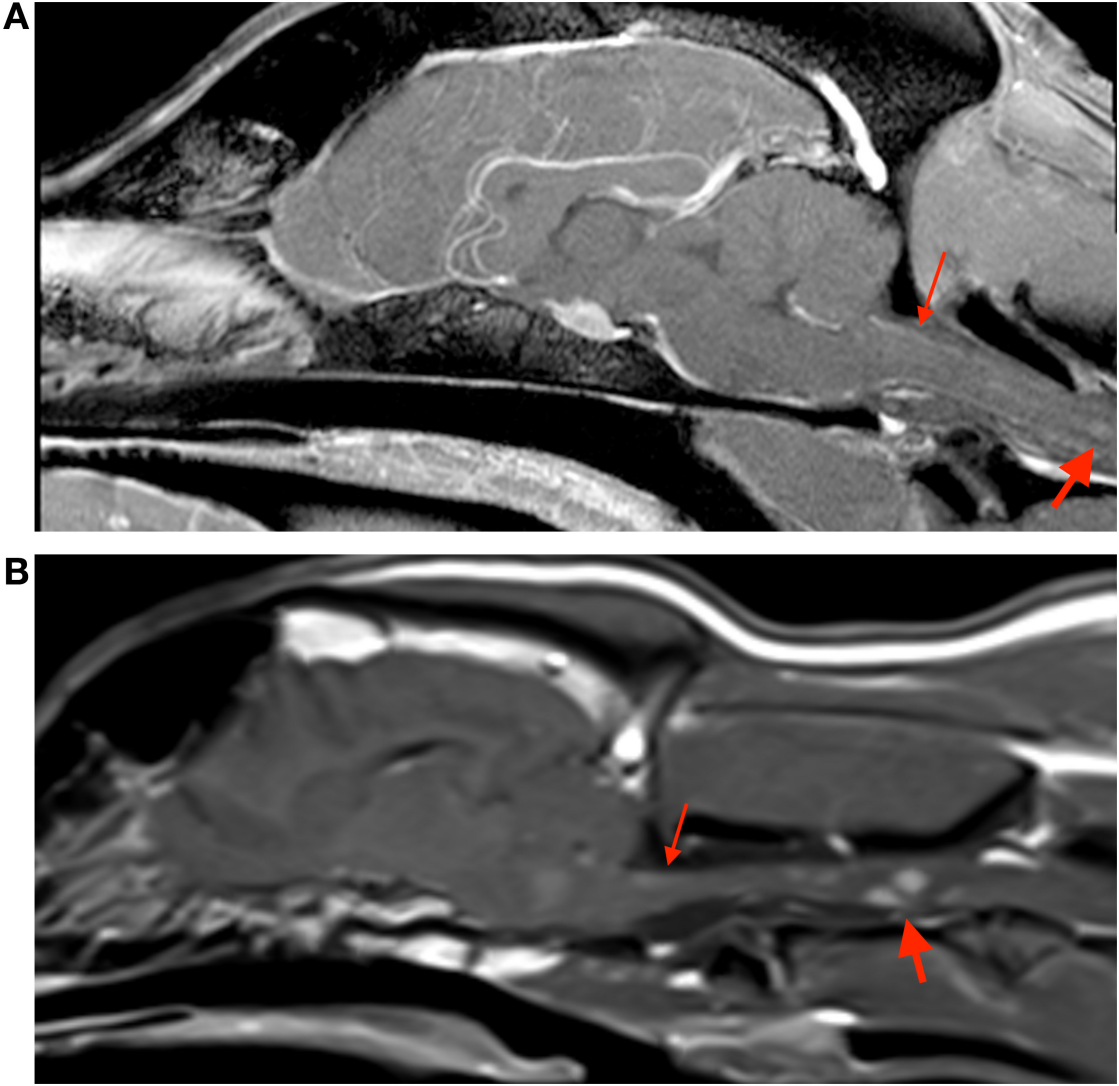
Sagittal post contrast t1-weighted images of the brain in case 1 **(A)** and case 3 **(B)**. Both images show small contrast enhancing lesions in the cervical spinal cord (thick arrows), and evidence of meningeal enhancement (thin arrows).

MRI abnormalities on pre contrast images were subtle and included faint regions of symmetric increased T2W and T2W-FLAIR signal intensity along the lateral aspect of the brainstem and enlargement of the optic (although subtle) and trigeminal nerves. Post contrast images revealed fairly symmetric contrast enhancement and/or enlargement of the optic, trigeminal, facial, and vestibulocochlear nerves (Figure 1, Case 1), as well as increased contrast enhancement in the orbital fissure (oculomotor, trochlear, ophthalmic branch of trigeminal, and abducens nerves). There was moderate diffuse leptomeningeal and pachymeningeal enhancement. There were also multifocal, poorly defined small intramedullary mildly contrast enhancing lesions within the cervical spine with evidence of meningeal enhancement (Figure 2A). Cisternal cerebrospinal fluid (CSF) analysis revealed a mononuclear pleocytosis (nucleated cell count of 160 cells/mm^3^, red blood cell count of 618 cells/mm^3^, and total protein of 181.5 mg/dL).

The patient was discharged with prednisone (0.5 mg/kg PO q24h), clindamycin (15 mg/kg PO q12h), meclizine (0.65 mg/kg PO q24h), and omeprazole (1 mg/kg PO q12h), as well as artificial tear ointment in the right eye 2–3 times a day due to facial nerve involvement (inability to fully close the right eye). Submitted infectious testing returned negative 6 days later (Neospora IFA, Toxoplasma IgG/IgM titers, and urine Blastomyces antigen). While CDV was a differential, it was not tested for due to its low incidence in geriatric dogs, up-to-date vaccination status, and clinical history atypical of this disease. Subsequently, her steroid dose was increased to twice daily administration, clindamycin was discontinued, and she was administered her first dose of cytarabine (200 mg/m^2^ IV over 24 h). Cytarabine was continued every 4 weeks subcutaneously (50 mg/m^2^ SC q2h for 4 doses).

The patient showed significant improvement in the following weeks. At the time of her 2 month recheck, her gait and mentation returned to normal, but she continued to have cranial nerve deficits, including incomplete menace response, absent lateral and diminished medial palpebral reflex OD. Her neurological examination remains unchanged at the time of writing, 5 months following her initial diagnosis.

### Case 2

A 10-year-old female spayed beagle (8.2 kg) was presented with a 2-week history of progressive difficulty walking, salivation, and a decreased appetite. Physical examination revealed blepharospasm and a corneal defect OD, but was otherwise unremarkable. Abnormal findings on the neurological examination included cerebellar and vestibular ataxia, right head tilt (30 degrees), miosis OD (attributed to a concurrent superficial corneal ulcer), absent menace response and palpebral reflex OD, and horizontal nystagmus (fast to the left). Postural reactions were delayed in the left thoracic limb and both pelvic limbs, and normal in the right thoracic limb. Withdrawal reflexes were normal in all limbs with diminished patellar reflexes (attributed to age-related change given concurrent hypertonia in all limbs). She was painful upon calvarial palpation without any paraspinal discomfort. A neuroanatomical localization was assigned to the brainstem and cerebellum. Differential diagnoses were the same as for case 1.

A Schirmer tear test was performed, and was within the normal reference range (22 mm/min) OU, and fluorescein staining confirmed a superficial corneal ulcer OD. Thoracic radiographs obtained prior to advanced imaging did not show any significant findings.

Clinical pathology abnormalities revealed a mild neutrophilia (12.77 [2.95–11.64 x 10^3^]) and relative lymphopenia (1.63 [1.05–5.10 x 10^3^]) consistent with a stress leukogram, and mildly elevated alkaline phosphatase likely secondary to stress. An Idexx SNAP 4Dx was also performed, and was negative for antibodies to Borrelia burgdorferi, Anaplasma phagocytophilum, and Ehrlichia canis, in addition to heartworm antigen.

Magnetic resonance imaging of the brain and the cranial cervical spine was performed using a 3T MRI system (Siemens Skyra; Siemens Medical Solutions, Malvern, PA, USA). MRI sequences included sagittal and dorsal T2W, transverse T2W, T1W, T2W-FLAIR, susceptibility weighted images, and post gadolinium-based contrast T1W sequences in three planes. Contrast medium (Omniscan® GE Healthcare Inc., USA) was administered intravenously at a dose of 0.1 mmol/kg.

Very similar as described for case 1, MRI abnormalities on pre contrast images were subtle and limited to regions of symmetric increased T2W and T2W-FLAIR signal intensity along the lateral aspect of the brainstem and enlargement of the optic (although subtle) and trigeminal nerves. Post contrast images revealed fairly symmetric contrast enhancement and/or enlargement of the optic, trigeminal, oculomotor, facial, and vestibulocochlear nerves, enhancement in the orbital fissure, and pachymeningeal and leptomeningeal enhancement (Figure 1, Case 2). Cisternal cerebrospinal fluid analysis revealed a mononuclear pleocytosis (TNCC of 18 cells/mm^3^, RBC of 3 cells/mm^3^, and a TP of 113.4 mg/dL). Infectious disease testing was submitted (Neospora IFA, Toxoplasma IgG/IgM titers, and urine Blastomyces antigen), all of which returned negative. Testing for CDV was not submitted due to the same rationale provided in case 1.

The patient was prescribed prednisone (1 mg/kg q24) and cytarabine (200 mg/m^2^ IV over 24 h), to be continued every 4 weeks subcutaneously (50 mg/m^2^ q2h for 4 doses). At her recheck 1 month later, her gait and head tilt (10–15 degrees) had improved, however she was noted to have bilateral facial nerve paralysis, and atrophy of the temporal muscle bilaterally. Due to this progression of cranial nerve signs, her cytarabine dose was increased to 225 mg/m^2^ (56 mg/m^2^ q2h for 4 doses) and she was started on Atopica (cyclosporine, 7.7 mg/kg PO q24h). She continued to receive this dose of cytarabine every 4 weeks as her prednisone was slowly tapered. Her cyclosporine dose remained unchanged.

At her last recheck 5 months following diagnosis, she was continuing to improve. Her gait and postural reactions were normal, however, she had a persistent mild left head tilt (5 degrees), and persistent facial nerve paresis (an incomplete menace bilaterally with ~25% closure and diminished palpebral reflex OU). Her neurological examination remains unchanged at the time of writing, 10 months following her initial diagnosis.

### Case 3

A 3 year-old female intact Australian Shepherd (19 kg) was presented for investigation of a 1-month history of a progressive left head tilt in spite of treatment for possible inner ear disease (antibiotics, steroids, and myringotomy). Physical examination was unremarkable. Abnormal findings on the neurological examination included vestibular ataxia, left sided circling, marked left head tilt, and rotary nystagmus (fast phase left). Postural reactions were delayed in all limbs, but worse on the left side. A neuroanatomical localization was assigned to the brainstem and cerebellum (central vestibular dysfunction). Differential diagnoses were the same as for case 1 and 2.

A complete blood count and serum biochemistry had been performed by the referring veterinarian, was reportedly normal but was unavailable for review at the time of writing of this manuscript.

Magnetic resonance imaging of the brain and cranial cervical spine was performed using a 1.5T system (Siemens Espree; Siemens Medical Solutions, Malvern, PA, USA). Sequences included sagittal T2W, transverse T2W, T1W, T2W-FLAIR, and T2^*^-weighted gradient recalled echo (GRE), diffusion weighted imaging, post contrast transverse T1-weighted thin section GRE images with fat suppression (“volume interpolated breathhold examination”; “VIBE”), and post contrast T1W spin echo sequences in three planes.

On pre-contrast images there were heterogeneous T2W hyperintensities in the cranial aspect of the pons and along the lateral portion of the brainstem, more extensive on the left, and subtle optic nerve enlargement bilaterally. A small intraparenchymal area of heterogeneous T2 hyperintensity and mild syringomyelia were seen in the cranial cervical spine. Following contrast medium administration there was variable degree contrast enhancement of the orbital fissure (oculomotor, trochlear, and abducens nerves) and the optic, oculomotor, trigeminal, facial, and vestibulocochlearnerves (Figure 1, Case 3). There was mild multifocal meningeal contrast enhancement as well as multifocal intramedullary and meningeal enhancement associated with the cranial cervical spine (Figure 2B). Cisternal CSF analysis revealed a marked mixed cell pleocytosis, which was predominantly lymphocytic (nucleated cell count of 304 cell/mm^3^, red blood cell count of 1 cell/mm^3^, and total protein of 202.7 mg/dL).

The patient was discharged with doxycycline (4 mg/kg PO q12h), clindamycin (12 mg/kg PO q12h), and prednisone (0.8 mg/kg PO q24h). She showed moderate improvement with decreased circling and an improved head tilt. Negative infectious tests were obtained 14 days later (Tick panel PCR, serology for Neospora antibodies, and Cryptococcus antigen latex). Testing for CDV was not submitted due to the same rationale provided in case 1 and 2. In turn, doxycycline and clindamycin were discontinued, and her prednisone dose was increased (to 1.6 mg/kg PO q24h).

At the time of her next recheck examination 2.5 months later, her neurological examination was unremarkable apart from a persistent mild left head tilt, and occasional circling to the left. Additional immunosuppressive medications were discussed, but declined at the time due to financial constraints. Additional recheck examinations have not been performed and the patient was lost to follow up.

## Discussion

This case series presents a novel presentation of MUE in 3 medium to large breed, female dogs with a similar pattern of bilateral cranial nerve involvement. All dogs presented with a progressive history of vestibular signs with either multifocal or central vestibular neurolocalization and multiple cranial nerve deficits. All three dogs were diagnosed with MUE based on previously proposed guidelines for antemortem diagnosis, meeting the following criteria: (1) older than 6 months with evidence of multifocal CNS disease on neurological examination or MRI, (2) hypercellular CSF analysis, (3) negative pertinent infectious testing, and (4) CSF exhibiting a mixed or lymphocytic pleocytosis (1, 8). To the author's knowledge, this is the first known case of antemortem diagnosis of MUE in a canine population documenting bilaterally symmetrical lesions affecting multiple cranial nerves.

The present population of dogs is not the typical demographic diagnosed with MUE. Most often this disease process affects young, small breed dogs with differing breed predilections depending upon the histopathologic diagnosis (1, 2, 8). The dogs in the present case series were larger and two were older than the average MUE patient, which has a mean age of presentation between <4 years (necrotizing encephalitides) and 4–8 years (GME), and a predilection for small and toy breed dogs (1, 2, 8). Females tend to be overrepresented, which is consistent with the current case series (2). While there is a significant amount of literature on MUE in the typical population, there is limited literature focusing on the disease in older larger dogs, with only one case series available (4). It is possible that larger and/or older dogs may develop a variation of MUE that is not encompassed by the well-documented patterns of MUE reported in small breed younger dogs. This case series adds to the limited literature of this disease presentation in the older and larger population of dogs.

Only two case reports have been published of dogs with a suspected immune mediated polyneuropathy including multiple cranial nerves, both diagnosed at necropsy. While optic neuritis is a known form of MUE, the optic nerves are a direct extension of the CNS, unlike cranial nerves III-XII which are classified as peripheral nerves (9). Fliegner et al. (10) described a 9-year-old Labrador Retriever that presented unable to close its mouth, with droopy lips, an altered bark, and mild pelvic limb ataxia. The dog has worsened despite treatment with an immunosuppressant dose of prednisone (0.9 mg/kg PO q12h). Electromyography of the masseter and distal limb muscles revealed increased insertional activity, fibrillation potentials and positive sharp waves, and CSF analysis revealed a mononuclear pleocytosis. Necropsy revealed granulomatous inflammation intra-axially in the brain and spinal cord, as well as in the peripheral nervous system, consistent with GME. Foss et al. (11) described a 4-year-old Golden Retriever that presented for investigation of a dropped jaw, mydriasis, difficulty swallowing, and a head tilt. Its neurological examination was consistent with autonomic, sensory, and motor involvement of multiple cranial nerves. Further testing including electrodiagnostics was declined and the dog was euthanized due to severity of disease. Necropsy revealed inflammatory lesions in the nerve fibers consistent with an immune mediated process, but no lesions were identified in the neuropil. Both of these cases were assigned a neuroanatomical localization to the peripheral neuromuscular system (PNS), and thus an MRI of the brain was not recommended or performed. The three dogs in this case series had examination findings consistent with lesions in both the central and peripheral nervous system, and MRI revealed lesions in both locations. It is interesting that MRI revealed more extensive lesions in the PNS compared to the CNS, despite central signs in all three dogs. While the MRI findings in the brainstem were more subtle than the peripheral lesions, more extensive CNS involvement cannot be excluded without histopathology, despite the use of a high field magnet (12). While the previous two cases were similar, this is the first publication documenting advanced imaging findings as well as a short term response to treatment in 3 dogs with MUE and a cranial polyneuropathy.

A combination of CNS and PNS signs have been reported in the humans with various autoantibody condition and as an uncommon manifestation of Guillain Barre Syndrome (GBS). Recently, cases have been described with PNS and CNS signs due to autoantibody encephalitides, including anti-AMPA and anti-NMDA receptor encephalitides, amongst other receptor targets (13, 14). A systematic review of human patients with patients with anti-glutamate receptor encephalitis found only 9/622 patients had PNS involvement, and the limbs appeared more commonly affected than the cranial nerves (14). Autoantibody encephalitides carry a variable prognosis depending on their ultimate target with cell surface neuronal antigens carrying a more favorable prognosis than intracellular neuronal antigens (15). Additionally, to the authors' knowledge, there have been no reported variants of autoantibody encephalitis restricted solely to the cranial nerves.

A rare variant of GBS referred to as polyneuritis cranialis has been reported to only affect the cranial nerves, accounting for 3–5% of Guillain Barre variant syndromes (16). Other variants of GBS involve a limited number of motor cranial nerves accompanied by limb numbness or weakness, such as multiple cranial neuropathy and Miller-Fisher syndrome (13, 16). Human patients lacking involvement of the extremities seem to have a considerably better prognosis than those with involvement of the cranial nerves and limbs. In a case series documenting 12 patients with a cranial nerve variant of GBS characterized by bilateral cranial nerve IX and X impairment and hypo- to areflexia of the limbs at onset, it was found to be rapidly progressive with numerous patients experiencing respiratory muscle paralysis (16). Four of these 12 patients required mechanical ventilation and one died within 24 h. Of the 11 patients that survived, recovery occurred over 1–3 months. A larger case series involving 61 individuals diagnosed with GBS (38 with cranial nerve involvement and 23 without) found a positive correlation between cranial nerve palsies and illness severity (17). All of the patients with cranial nerve involvement developed such marked quadriparesis they were bed bound. Additionally, significantly more patients with cranial nerve involvement required ventilatory support compared to GBS patients lacking cranial nerve involvement, being 52.6 and 8.7%, respectively. Two patients requiring respiratory support died. Guillain Barre syndrome is thought to have a similar pathogenesis as canine polyradiculoneuritis. While both of these disease process can affect multiple cranial nerves, this is due to a generalized polyneuropathy that is limited to the PNS with no CNS involvement (18). Since the three cases in this series all had evidence of CNS involvement, this likely presents a different disease entity.

The cases described here may represent a variation of MUE yet to be described. Similar to polyneuritis cranialis in humans, clinical improvement was seen after implementing immunomodulatory therapy in all three dogs. The most significant clinical improvement occurred over the first 2 months of treatment with immunosuppressant prednisone (3/3) and cytarabine (2/3), and all were alive at the time of writing. Multiple studies evaluating the survival factors of dogs diagnosed with MUE, have shown that survival at 3 months is strongly predictive of survival at 1 year (19–21). Thus, dogs with this variation of MUE may have a better prognosis than indicated by the two previous published cases diagnosed only post-mortem. However, none of these dogs had a complete resolution of their cranial nerve signs, with a persistent head tilt and/or facial nerve paresis in all three dogs. Interestingly, a case series including 26 dogs with idiopathic trigeminal neuropathy documented 100% recovery (22), while another series of 16 dogs with concurrent facial and vestibular neuropathy only found 31% of dogs to have a complete resolution of signs (23). The majority of dogs with bilateral trigeminal neuropathy are reported to be idiopathic in origin, while unilateral trigeminal neuropathies can be neoplastic, inflammatory neuritis, or less often idiopathic (24). It has been proposed that concurrent facial and vestibular neuropathy may be inflammatory in origin, however, in one case series of 16 dogs, none had elevations in their total nucleated cell count on CSF analysis (23). Additionally, recovery in these cases appeared to be independent of treatment administered, including if no treatment was administered (22, 23). Since trigeminal, facial, and vestibular neuropathies are generally not life-limiting, none of the dogs in these case series had histopathological analysis, thus it is difficult to conclude whether they have an inflammatory element, and if there is another disease process present in those diagnosed with “idiopathic” neuropathies. While we may not fully understand the underlying etiologies for these diseases, outcome data available indicates that the facial and vestibulocochlear nerves may have a worse prognosis for return to function compared to the trigeminal nerves, as was observed in these 3 cases.

The major MRI abnormalities seen in our patient population were bilateral variable severity contrast enhancement and enlargement of the optic, trigeminal, oculomotor, facial, and vestibulocochlear nerves, the orbital fissure, as well as the meninges of brain and cranial cervical spine. It is interesting that the abnormal cranial nerves identified on MRI did not always correlate with the deficits on neurological examination. While all dogs had dysfunction of the facial and vestibulocochlear nerves, trigeminal deficits were only detected in 1/3 dogs, despite trigeminal nerve enlargement seen on MRI in all three dogs. The two previous similar case reports of suspected immune mediated polyneuropathy including multiple cranial nerves (10, 11) also had involvement of the glossopharyngeal and vagus nerves indicated by clinical signs. While the three cases presented here did not have neurological deficits indicating involvement of other cranial nerves, many of the other cranial nerves can be difficult or impossible to visualize on MRI (25). It is possible that smaller slices in our MRI protocol may have allowed visualization of abnormalities of cranial nerves 9, 10, and 11, although the origins of cranial nerves 6 and 12 are notoriously difficult to visualize on MRI (25, 26). This information highlights the importance of thin-slice studies when evaluating the cranial nerves.

In summary, this case series presents MRI findings, clinical response, and short term survival in three dogs with MUE also involving multiple cranial nerves. More cranial nerves were affected than were initially suspected based on their preliminary neurological examination in all three dogs. These cases serve as a reminder that when a cranial nerve polyneuropathy is detected, MRI and CSF analysis remain strongly indicated, as lesions can be in the CNS, PNS, or both. In MUE cases with multiple cranial nerve involvement, immunosuppressant medications may be helpful, however, owners should be warned that cranial nerve signs may persist or take longer to resolve. This case series documents the first ante-mortem diagnosis and response to treatment of this novel variation of MUE with multiple cranial nerve involvement.

## Ethics Statement

Ethical review and approval was not required for the animal study as it is not required for retrospective case reports. Written informed consent was obtained from owners for participation of their animals.

## Author Contributions

HL helped with diagnosis and treatment of patients 1 and 2, and SH recruited patient 3. HL and RL drafted the initial manuscript in collaboration with SH. The MRI images were reviewed and interpreted by HL, RL, and SH. All authors contributed to the manuscript final revision and have read and approved the submitted manuscript.

## Conflict of Interest

The authors declare that the research was conducted in the absence of any commercial or financial relationships that could be construed as a potential conflict of interest.

## Abbreviations

CNS: central nervous system
MUE: meningoencephalitis of unknown etiology
GME: granulomatous meningoencephalitis
GBS: Guillain Barre syndrome
NME: necrotizing meningoencephalitis
NLE: necrotizing leukoencephalitis
MRI: magnetic resonance imaging
CSF: cerebrospinal fluid
T1W: T1-weighted
T2W: T2-weighted
OU: both eyes
OD: right eye
PNS: peripheral nervous system.
